# A two-step tilt compensation method for off-axis holographic displays

**DOI:** 10.1038/s41598-026-55232-2

**Published:** 2026-07-15

**Authors:** Roubing Meng, Antoni J. Wojcik, Zhongling Huang, Timothy D. Wilkinson

**Affiliations:** Electrical Engineering Division, Department of Engineering, University of Cambridge, Cambridge, CB3 0FA UK

**Keywords:** Angular spectrum method, Holographic display, 3D display, Engineering, Optics and photonics, Physics

## Abstract

Accurate diffraction between non-parallel planes is essential for holographic displays employing tilted spatial light modulators (SLMs). However, applying the standard angular spectrum method (ASM) directly to a tilted plane leads to geometric distortion and spectral aliasing. We present a two-step propagation method and its validation by experiments. The proposed method combines the standard ASM with a spatial-domain rotational transformation. The target image is first back-propagated to a parallel intermediate plane, then geometrically remapped onto the tilted SLM with phase correction. Implemented on an off-axis holographic display with $30^\circ$ and $45^\circ$ tilted SLM which leads to a large steering angle between the incident and reflected beam, the method is evaluated with multi-depth and multi-angle reconstructions. Results show improved image fidelity, stable depth performance, and robust compensation of off-axis holographic displays.

## Introduction

Holographic displays are considered as a promising next-generation imaging and visualization technology because they can reconstruct the full information carried by a wavefront from an object, offering natural depth cues and continuous parallax. Their ability to generate true three-dimensional (3D) image without the need for extra wearable devices has driven interest across a wide range of applications, including augmented and virtual reality (AR/VR) near-eye displays^1^, full-color 3D visualization systems^2^, automotive head-up displays^3^, optical trapping and beam shaping^4,5^, zoom function and miniaturization of meta-holography ^6,7^, and medical imaging^8^. As these applications demand wider viewing angles, higher resolutions, and more compact optical geometries, accurate numerical light propagation becomes essential for generating high-quality reconstructions.

In far-field propagation, also known as Fraunhofer diffraction, the relationship between the object field and the diffracted field can be approximated to a Fourier transform. Most phase-retrieval two-dimensional (2D) algorithms, such as the Gerchberg–Saxton (GS)^9^ algorithm and the One-Step Phase Retrieval (OSPR) algorithm^10^, operate the fast Fourier transformation (FFT) to get far-field diffraction. Because these algorithms do not depend on propagation distance, they can be applied to both parallel and non-parallel configurations without modification, and geometric tilt does not directly affect their reconstruction qualities. However, many practical holographic display systems reconstruct 3D objects in the near field, where propagation need to use distance-dependent diffraction formulations, such as the Fresnel diffraction^11^ and the angular spectrum method (ASM)^12^. In these methods, the propagation distance plays a critical role in generating holograms and reconstruction accuracy. Most near-field algorithms assume that the source and destination planes are parallel, and are not applicable between non-parallel planes. However, as holographic display architectures become more immersive and complex, non-parallel design is more common in the configurations, such as multiple SLMs^13–15^, multiple laser pathways^2,16^, and polygon-based computer-generated holography (CGH)^17^. In these cases, applying algorithms without considering the non-parallel propagation leads to sampling inconsistencies, distortion, and spectral aliasing, resulting in poor reconstruction quality.

Research into hologram generation on tilted planes has gained significant attention in recent years as holographic displays increasingly adopt non-parallel optical geometries. Matsushima proposes a frequency-domain rotational transformation based on angular spectrum method^18,19^. However, their approach implements the rotational transformation in the Fourier domain and the interpolation on a frequency grid as well as requires additional Jacobian compensation, which increase numerical complexity and make the method less straightforward to integrate with standard ASM. Kozacki investigates the holographic display with a tilted LCoS SLM and proposes a plane-wave spectrum (PWS) remapping algorithm for tilted SLM geometry^20^. Similar to the method proposed by Matsushima^18,19^, they both implements the remapping and interpolation in Fourier domain. Moreover, the algorithm proposed by Kozacki is based on Fresnel propagation rather than an angular spectrum which is restricted to paraxial fields. Chang et al. have explored two distinct numerical approaches for tilted-plane diffraction. One work introduces a nonuniform fast Fourier transformation (NUFFT) based on angular-spectrum framework capable of handling the non-uniform spectral sampling caused by Fourier-domain rotation, enabling scalable hologram computation with variable sampling rates and avoiding the interpolation required in conventional FFT-based rotated ASM methods^21^. Their later work employs the fractional Fourier transform (FRFT) to derive a more accurate tilted-plane diffraction model aimed at reducing approximation error for moderate rotation angles^22^. While both approaches demonstrated the ability of calculating diffraction between tilted planes, they integrate NUFFT and FRFT operators with iterative hologram generation algorithms which increases computational and implementation complexity. Harm et al. demonstrated that liquid-crystal SLMs with extended phase depth can multiplex holograms for different readout angles by exploiting angle-dependent phase-wrapping, but their method relies on device-specific calibration and is not directly applicable to general CGH propagation problems^23^. More recently, Xia et al. incorporate stochastic gradient descent (SGD) with spectrum remapping to generate off-axis holograms while utilizing the camera-in-the-loop (CITL) to iteratively update the holograms by minimizing a loss function^24^. Because this approach integrates spectrum remapping within each iteration of the optimization loop, it incurs a heavy computational cost.

This paper presents a simple method for performing tilted-plane propagation based on the angular spectrum framework for off-axis holographic displays and its experimental validation. The method decomposes the problem into a two-step process: (i) a standard backward ASM from the target screen to a parallel intermediate plane, and (ii) a rotational transformation in the spatial domain to compute the field on the tilted SLM with phase compensation. We implement the proposed method on an off-axis holographic display setup and evaluate its performance through interpolation analysis, comparison with standard ASM, multi-depth reconstruction, tolerance to SLM tilt angle variations, and complex image reconstructions. The results demonstrate that the method provides accurate reconstruction across depth and angle while preserving computational simplicity.

This paper first presents the principle and both numerical and experimental implementations of the proposed two-step tilted-plane compensation method, followed by experimental results under different configurations. Finally, the reconstruction performance, tolerance to variable configurations and limitations of the method are discussed.

## Principle and implementation

### Standard angular spectrum method

Light propagation between two parallel planes can be accurately computed using the ASM. Starting from the Rayleigh-Sommerfeld diffraction integral, the field at a distance *z* from the source distribution *g(x',y',0)* can be expressed as^12^:

1 $$\begin{aligned} g(x,y,z) = \frac{1}{i\lambda }\iint g(x',y',0)\frac{e^{ikr}}{r}\frac{z}{r}\left( 1+\frac{i}{kr}\right) \textrm{d}x'\textrm{d}y' \end{aligned}$$

where $r=\sqrt{(x-x')^2+(y-y')^2+z^2}$ and $k=\frac{2\pi }{\lambda }$ is the wavenumber. The integral can be rewritten as a convolution of the light field at the original plane with a propagation kernel *h*(*x*, *y*, *z*) as:

2 $$\begin{aligned} g(x,y,z) = g(x',y',0)*h(x,y,z) \end{aligned}$$

where the propagation kernel is:

3 $$\begin{aligned} h(x,y,z) = \frac{1}{i\lambda }\frac{e^{ikr}}{r}\frac{z}{r}\left( 1+\frac{i}{kr}\right) \end{aligned}$$

By taking the Fourier transform for both sides of Eq. (2), the convolution becomes a multiplication:

4 $$\begin{aligned} G(u,v;z) = G(u,v;0)H(u,v;z) \end{aligned}$$

and the field at distance *z* is obtained by:

5 $$\begin{aligned} g(x,y,z) = \mathcal {F}^{-1}\left\{ G(u,v;0)H(u,v;z)\right\} \end{aligned}$$

This forms the standard ASM framework used in hologram generation. However, the standard ASM is not directly applicable to holographic display configurations with a tilted SLM, as it lead to geometric distortion and degraded reconstructions.

### Optical setup

**Fig. 1:**
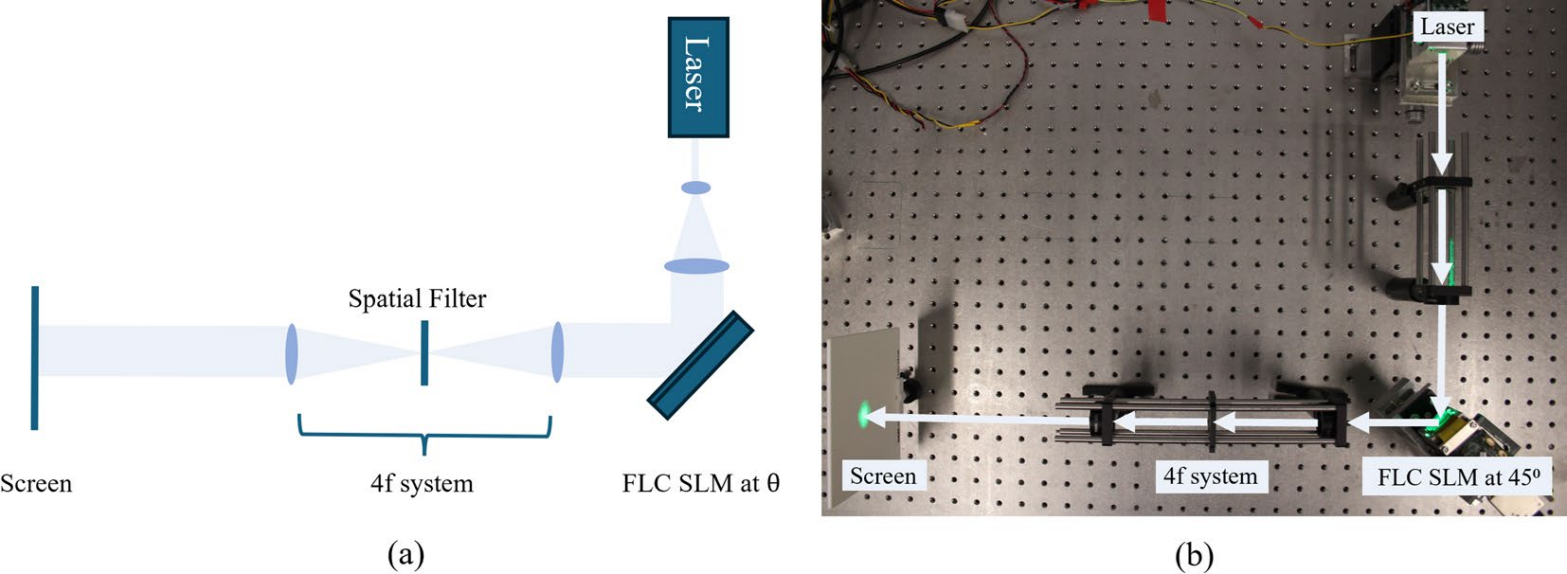
(**a**) The schematic of the optical setup. (**b**) The experimental setup with a SLM tilted at $45^\circ$.

Before introducing the proposed method to compensate the tilted SLM configuration, the optical setup is presented first as shown in Fig. 1. The schematic of the optical setup is shown in Fig. 1a. The SLM is tilted with respect to both the optical axis and to the screen at an angle of *θ*. Here, the term “off-axis” refers to the tilted incidence of the illuminating light on the SLM. Then the modulated beam passes through a 4f system where a spatial filter placed at the Fourier plane to eliminate the zero-order noise. The filtered beam is then projected onto a screen located at a distance of *z* from the conjugate SLM image formed by the 4f system. The 4f system is assumed to operate as an ideal unit-magnification relay. Therefore, the complex optical field at the physical tilted SLM is reproduced at a virtual conjugate plane after the 4f system, which is denoted as SLM*. As the relay imaging preserves the complex field between conjugate planes, the 4f system does not introduce additional diffraction effects.

The experimental setup is shown in Fig. 1b. An RGB laser is expanded and collimated before illuminating the ferroelectric liquid crystal (FLC) SLM (Forth Dimension Displays SXGA-R2D^25^). The SLM has a resolution of *1280 × 1024* pixels with a pixel pitch of $13.62 \upmu \,\hbox {m}$. The SLM is tilted by an angle of $\theta = 45^\circ$ relative to the incident beam, resulting in an angle of $90^\circ$ between the incident and reflected beams. A 4f filtering system consisting of two lenses with focal lengths $f_1=f_2 = 10\,\hbox {cm}$ is placed after the SLM and a physical screen is placed at a distance of *z*.

### Implementation of two-step tilt compensation

**Fig. 2:**
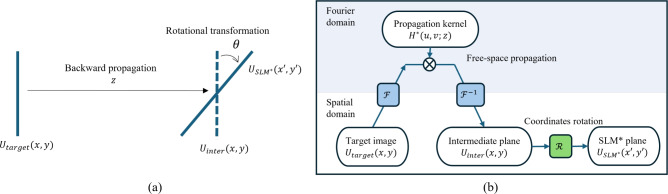
Two-step tilt compensation method. (**a**) Geometry of planes. (**b**) The flowchart of the two-step numerical implementation.

Propagation between two parallel planes can be efficiently computed using the standard ASM, as described earlier in this section. In an off-axis holographic display setup, as shown in Fig. 1a, directly applying the standard ASM to this non-parallel geometry results in geometric distortions and aliasing artifacts. To accurately calculate the hologram pattern on the SLM* plane, a two-step backward propagation algorithm based on the ASM and a local rotational transformation is proposed as shown in Fig. 2. Fig. 2a illustrates the geometrical relationship between planes involved in the proposed algorithm. The target image is the screen plane of the optical setup in Fig. 1 which is located at a distance *z* from the center of the tilted SLM* and is parallel to the optical axis while the SLM* plane is tilted at an angle of *θ*. An intermediate plane is parallel to the screen and positional at the same axial distance *z* as the SLM* center.

Figure 2b presents the flowchart of the numerical implementation of the proposed two-step method. Step 1 is a backward angular spectrum propagation from the target image $U_{target}(x, y)$ to the intermediate plane $U_{inter}(x,y)$. Since the screen and the intermediate plane are parallel, the standard backward ASM can be used according to Eq. (5):

6 $$\begin{aligned} U_{inter}(x,y) = \mathcal {F}^{-1}\left\{ \mathcal {F}\left\{ U_{target}\right\} \cdot H^*(u,v;z)\right\} \end{aligned}$$

where $H^*$ is the transfer function for backward propagation. Step 2 is a rotational transformation in spatial domain to calculate the field distribution on the tilted SLM* plane. The intermediate plane is defined as the origin of the local coordinate system to simplify the coordinate remapping. The coordinate transformation between the intermediate plane (*x*, *y*) and the tilted SLM* plane *(x',y')* with a tilt angle *θ* around the y-axis is:

7 $$\begin{aligned} \begin{aligned} x'&=x \cos {\theta } \\ y'&=y \end{aligned} \end{aligned}$$

The tilt also introduces an additional optical path difference $z'=x\sin {\theta }$. A linear phase term $\exp {(ikx'\sin {\theta })}$ is compensated. The final implementation to calculate the hologram on the tilted SLM* is:

8 $$\begin{aligned} U_{SLM}(x',y') = U_{inter}(x'=x \cos {\theta },y'=y)\cdot \exp {(ikz')} \end{aligned}$$

which is equivalent to the hologram on the physical tilted SLM.

Moreover, the rotational transformation maps the complex field onto non-integer spatial coordinates, which do not locate on the discrete sampling grid of the SLM. Therefore, interpolation is required to resample the field onto the SLM pixels. In this work, we firstly employed and compared several interpolation methods to maintain smooth transitions and avoid aliasing.

## Results

In this section, we experimentally validate the proposed method on the off-axis holographic display and analyze its performance under different conditions. We first compare several interpolation methods used in the rotational mapping to find the best interpolation method as a default in the sequential experiments. We then compare the reconstructed results obtained by the proposed method and by the standard ASM. Finally, we applied the proposed method to multi-depth and different tilt-angle configurations by examining the reconstructions at different propagation distances and under different SLM tilt angles.

### Interpolation methods analysis

**Fig. 3:**
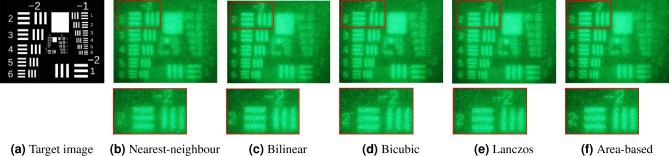
(**a**) Target image. (**b**)–(**f**) The results are reconstructed at $50\,\hbox {cm}$ with different interpolation methods.

To optimize the reconstruction quality, five distinct interpolation methods were evaluated: nearest-neighbour, bilinear, bicubic, area-based interpolation^26^ and Lanczos method^27^. A USAF resolution chart^28^ was used as the test pattern and all images were reconstructed with the tilt SLM at an angle of $45^\circ$ and propagated at a distance of $50\,\hbox {cm}$. The performance of each method was assessed both by visual inspection of the reconstructed images as shown in Fig. 3 and quantitative evaluation as shown in Table 1.

As illustrated in Fig. 3(**b**)–(**f**), all interpolation methods are able to reproduce the general pattern and exhibit very similar visual quality with subtle differences observable by visual inspection. To enable an objective comparison, quantitative image quality metrics are therefore used to evaluate the reconstruction quality of each method. Table 1 provides a quantitative assessment using three standard metrics: Normalized Mean Square Error (NMSE), Peak Signal-to-Noise Ratio (PSNR), and Structural Similarity Index Measure (SSIM)^29^. The bilinear interpolation shows the best performance with the lowest NMSE and the highest PSNR and SSIM, while the bicubic method gives an acceptable performance. In particular, the area-based method performed poorly in this experimental setup, showing the worst results for most metrics.

**Table 1:** Quantitative evaluation for different interpolation methods.

Methods	Nearest-neighbour	Bilinear	Bicubic	Lanczos	Area-based
NMSE	1.546	1.341	1.371	1.563	1.669
PSNR	6.69	7.31	7.21	6.64	6.36
SSIM	0.168	0.407	0.112	0.124	0.116

These results suggest that interpolation choice has a measurable effect on reconstruction quality. However, the differences remain subtle in visual appearance due to the dominant influence of optical noise and speckle in the experimental system. Among all tested methods, bilinear interpolation provides the most consistent performance across all metrics, and is therefore used as the default method in the sequential experiments.

### Comparison with standard ASM

**Fig. 4:**
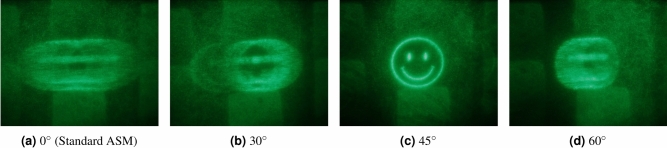
Reconstruction results at a distance *z* of $50\,\hbox {cm}$. (**a**) Reconstruction obtained using the standard ASM without rotational transformation. (**b**)–(**d**) Reconstructions obtained with rotational transformation angles of $30^\circ$, $45^\circ$ and $60^\circ$ in the hologram generation, while the physical tilt angle of the SLM is fixed at $45^\circ$. When the compensation angle does not correspond to the physical tilt angle, the reconstructions remain unclear and distorted due to geometric mismatch.

After setting the default interpolation method, we evaluated the efficacy of the proposed method. The setup tested in this section has the SLM physically tilted at an angle of $\theta = 45^\circ$ relative to the optical axis. The hologram was calculated for a target image positioned at a distance of $z = 50\,\hbox {cm}$ from the SLM* plane. The results are shown in Fig. 4 by varying the rotation angle parameter *θ* used in the hologram generation, while keeping the physical tilted angle of the SLM fixed at $45^\circ$. Figure 4a corresponds to the standard ASM without rotational transformation which means the angle of the rotational transformation is $0^\circ$ in the hologram generation, whereas Fig. 4b–d show the results obtained when rotation angles of $30^\circ$, $45^\circ$, and $60^\circ$ are applied.

As seen in Fig. 4a, without rotational transformation the reconstructed pattern appears stretched and distorted due to the mismatch between the parallel-plane assumption of ASM and the tilted SLM geometry. When a rotation angle of $30^\circ$ is applied, the distortion is partially corrected but remains visible, indicating under-compensation relative to the physical SLM tilt. When the rotational transformation matches the physical tilt of the SLM which is $45^\circ$, the output becomes significantly clearer and sharper, as shown in Fig. 4c. Increasing the rotation angle beyond the physical tilt to $60^\circ$ introduces distortion again as shown in Fig. 4d, corresponding to over-compensation. This progression confirms that the proposed spatial transformation compensates the tilted geometry in a physically consistent manner and that accurate matching between the physical SLM tilt and the algorithmic rotation angle is essential for optimal reconstruction.

### Depth stability and tilt angle tolerance

**Fig. 5:**
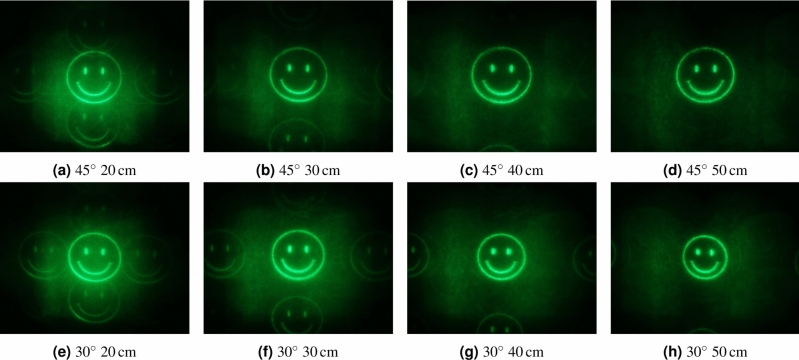
Reconstructions with different experimental configurations. (**a**)–(**d**) The SLM is physically tilted at $45^\circ$ while the screen is located at $20\,\hbox {cm}$, $30\,\hbox {cm}$, $40\,\hbox {cm}$ and $50\,\hbox {cm}$. The holograms are generated with rotation angle of $45^\circ$. (**e**)–(**h**) The SLM is physically tilted at $30^\circ$ while the screen is located at $20\,\hbox {cm}$, $30\,\hbox {cm}$, $40\,\hbox {cm}$, and $50\,\hbox {cm}$. The holograms are generated with rotation angle of $30^\circ$. As the propagation distance increases, the faint secondary replicas move farther away and eventually go outside of the screen region at the propagation distance of $50\,\hbox {cm}$.

We then evaluated the robustness of the proposed method under different experimental configurations to validate its depth stability and tolerance to variations in the SLM tilt angle. Figure 5 illustrates the reconstructions with different experimental configurations. For Fig. 5a–d, the SLM was physically tilted at $45^\circ$ and the corresponding rotational correction angle was used in the hologram generation. The propagation distance *z* was varied from 20 to 50 cm. For Fig. 5e–g, the SLM was physically re-tilted to $30^\circ$ and the rotational angle used in the hologram generation was updated accordingly. The propagation distance *z* was varied from 20 to 50 cm.

As shown in Fig. 5, the reconstructed images remain clear and focused across all tested propagation distances when the appropriate tilt compensation is applied. No noticeable increase in geometric distortion or loss of sharpness is observed as *z* increases within this range, indicating that the proposed method maintains stable reconstruction quality across depth. In addition, consistent reconstruction quality is observed for different physical SLM tilt angles when the corresponding rotation angle is applied, demonstrating that the proposed method is not restricted to a single tilt configuration and can compensate for varied off-axis geometries. As the propagation distance *z* increases, the primary reconstructed image remains clear and sharp, with no broadening of the image, indicating that the lateral resolution is preserved across the tested depth range. At smaller propagation distances, faint secondary replicas can be observed around the primary image. As the propagation distance *z* increases, these replicas move farther away and eventually go outside of the screen region. These replicas are high-order diffracted components caused by the finite pixel pitch and size of the SLM and do not degrade the quality of the primary reconstructions.

These results indicate that the proposed tilted-plane compensation corrects the geometric mismatch introduced by the off-axis configuration and provides stable reconstruction performance over a range of propagation distances and SLM tilt angles supporting its application in off-axis 3D holographic display systems.

### Reconstructions of complex images

**Fig. 6:**
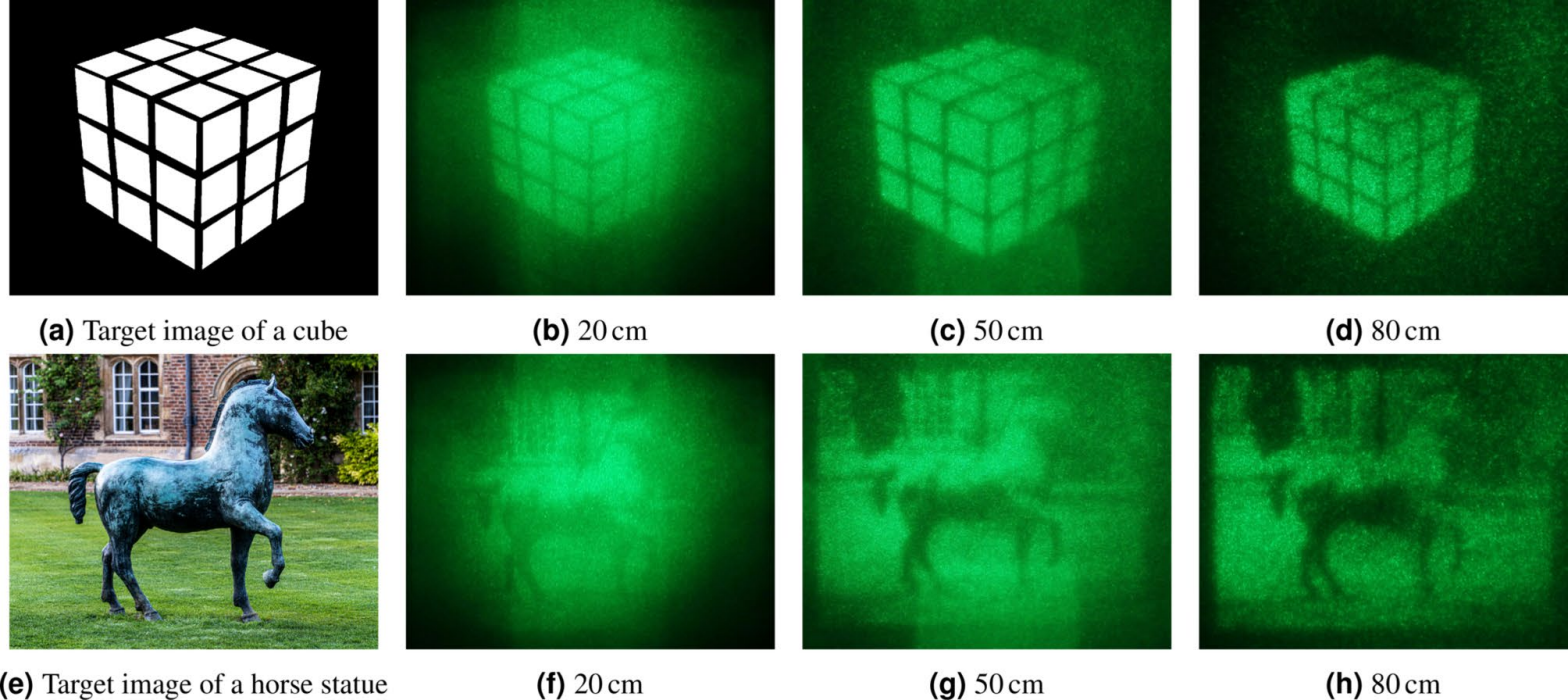
(**a**) Target image of a cube. (**b**)–(**d**) Reconstructions of the cube at $20\,\hbox {cm}$, $50\,\hbox {cm}$, and $80\,\hbox {cm}$. (**e**) Target image of a horse statue. (**f**)–(**h**) Reconstructions of the horse statue at $20\,\hbox {cm}$, $50\,\hbox {cm}$, and $80\,\hbox {cm}$.

To further validate the proposed method beyond simple patterns, we extended our evaluation to include complex, grayscale target images. Figure 6 displays the reconstructions of a cube and a horse statue at varying propagation distances of $20\,\hbox {cm}$, $50\,\hbox {cm}$, and $80\,\hbox {cm}$. The results confirm that our method successfully preserves the structural integrity and geometric accuracy of complex and grayscale targets across the tested depth range.

While the reconstructed images exhibit limited resolution which is constrained by the finite pixel pitch ($13.62\upmu \,\hbox {m}$) and the binary phase modulation of the FLC SLM, the successful reproduction of complex grayscale target images demonstrates that the proposed compensation method is not limited to simple geometric patterns. By accurately modeling light propagation between non-parallel planes, the method maintains structural integrity for complex target images. Further improvements could be achieved by utilizing SLMs with higher resolutions or by introducing iterative methods into the algorithm.

## Discussion and limitations

The experimental results presented in previous sections demonstrate that the proposed tilted-plane propagation method overcomes the geometric limitations of applying the ASM to non-parallel planes. The distorted reconstruction observed in the Fig. 4a generated by the standard ASM is caused by the mismatch between the tilted plane and the parallel-plane assumption of the standard ASM. By incorporating a rotational coordinate transformation that matches the physical tilt angle of the SLM, the proposed method corrects this mismatch and reconstructs clear images.

The results of multi-depth and multi-angle configurations in Fig. 5 confirm that the reconstructed images remain stable and well-focused across a depth range and a tilt angle range. This is useful in multi-plane and multi-path holographic display configurations which are commonly used to extend the field of view. For example, in wide-angle holographic displays with multi-SLM configurations^13,14^, each SLM reconstructs a portion of the overall replay field along a distinct optical path from a different angle. The proposed method can be used to eliminate the distortion caused by the complex geometry and improve the quality of the reconstruction. In addition, the ability to maintain consistent quality across depth validates the feasibility of the approach offers depth cues and volumetric perception within a compact optical system.

The experimental results validate that the method performs reliably at a large physical SLM tilt of $45^\circ$, corresponding to a $90^\circ$ angle between the incident and reflected beams. The tolerance to large tilt angles provides additional flexibility for compact optical design, enabling larger angular separation and facilitating more versatile system layouts. In reflective display geometries, such a steering angle is sufficient to achieve effective spatial separation between illumination and observation paths and to integrate more compact optical layouts. From a theoretical perspective, the proposed compensation method does not have an upper bound on the compensation angle, and larger tilt angles could be handled through the same rotational transformation. However, constrains such as beam overlap and additional alignment complexity limit the practical tilt angle range. The tolerance to large tilt angles provides additional flexibility for compact optical design, enabling larger angular separation and facilitating more versatile system layouts.

The sensitivity of the reconstruction quality to angular mismatch between the physical SLM tilt and the angle used in the coordinate transformation is an important consideration. As observed experimentally, increasing mismatch leads to progressive degradation of image quality. While a systematic quantitative tolerance analysis is beyond the scope of this work, the results indicate that accurate calibration of the SLM tilt is required for optimal performance. The tolerable angular error is expected to depend on both the propagation distance and the effective spatial bandwidth of the hologram, with larger distances and higher spatial frequencies leading to increased sensitivity.

Despite the demonstrated robustness, certain limitations remain. Blurring in high-frequency regions of the reconstructed images is observed, which is primarily caused by coherent noise, finite pixel pitch and low resolution of the SLM. In addition, faint replica patterns which are higher diffraction orders are observed in the reconstructed images, particularly at short propagation distances. At larger propagation distances, these higher-order components diverge spatially and are effectively separated from the main reconstruction, resulting in their reduced visibility. However, the near propagation distance can cause partial overlap between the primary reconstruction and higher diffracted orders, making these replicas more noticeable. Furthermore, while this study assumes the 4f system functions as an ideal, unit-magnification relay. In reality, the 4f system with a tilted image plane can introduce additional phase errors and geometric distortions that influence reconstruction quality. Therefore, further analysis of these imperfections would be valuable for further studies.

Overall, the proposed tilted-plane compensation method provides a practical and computationally efficient solution for off-axis holographic displays, effectively correcting geometric distortion introduced by non-parallel propagation while maintaining reconstruction stability across depth and angle. Future work will extend the method to full-color reconstruction, incorporate hologram optimization techniques for speckle suppression and image quality improvement.

## Conclusion

In this work, we have proposed and experimentally validated a practical method for calculating holograms on tilted SLM planes in off-axis holographic displays. By combining the standard backward ASM with a spatial-domain rotational transformation and phase correction, the proposed approach effectively addresses the geometric distortion and spectral aliasing caused by the standard ASM when applied to non-parallel propagation geometries. Experimental results demonstrate that the proposed method reconstructs images with improved quality compared to the standard ASM. Furthermore, the method exhibits stable performance across a range of propagation distances and variable SLM tilt angles, confirming its robustness for practical off-axis display configurations. The reconstructed images remain well-focused over varying depths and angles.

The proposed tilted-plane compensation method provides an efficient and straightforward solution for off-axis holographic displays, enabling improved reconstruction quality in compact and wide-angle holographic systems such as multi-SLM displays, wide-angle near-eye displays, and other compact holographic architectures employing non-parallel optical geometries.

## Data Availability

Data sets generated during the current study are available from the corresponding author on reasonable request.
